# Report of a Case of Intra-Uterine Polypus, with Operation

**Published:** 1867-02

**Authors:** W. F. Westmoreland

**Affiliations:** Professor of Surgery in the Atlanta Medical College


### ARTICLE III.

*Report of a Case of Intra-Uterine Polypus, with Operation.* By W. F. WESTMORELAND, M. D., Professor of Surgery in the Atlanta Medical College.

We do not propose, in this article, to discuss the different varieties of uterine polypi, or the several operations that have been suggested for their removal, but simply to give the report of a case illustrating a simple, and to me, a new method of removing certain varieties of *intra-uterine* polypi. No one who has been called upon to treat a case of this most perplexing affection, in a patient exhausted to the last degree, by repeated hemorrhages, but will be interested in the suggestion of a more simple and less hazardous operation than has usually been adopted in the removal of this class of uterine out-growths.

On the 12th of September, 1866, I saw, with Drs. Shaffee and Talley, Mrs. K——, of Forsyth county, Georgia, who, for five or six months, had suffered from an exhausting uterine hemorrhage. I found a rather delicate lady, 34 years of age, who, for the past ten years, had suffered greatly from indigestion.

She informed me that she had had five children, the youngest two years old; never had any uterial trouble until since the birth of her last child. In March last, fifteen months after the birth of her last child, and about her usual time for the return of the menstrual flow after child-birth, she had her first hemorrhage, but as it was not profuse, she regarded it as nothing more than the return of the natural discharge. In a short time after the first, she had a second hemorrhage, more profuse; since which time they had rapidly increased in frequency and quantity. At the time of my visit the hemorrhage was almost continuous, the patient confined to the bed, and exsanguinous to the last degree.

Sometime before my visit, the physicians in attendance had detected a tumor within the cervi uterix, and it was for the removal of this that my services were demanded.

Upon an examination per vagina, I had some difficulty in reaching the tumor, while the patient occupied the recumbent position; but as soon as she was placed in the half-sitting posture, I had no difficulty in detecting the polypus, but found it would be impossible to introduce my finger in the uterus, as this organ would recede before the force necessary to pass the cervix. Feeling the necessity of the immediate removal of the polypus, and believing that the os uteri was sufficiently dilated to readily permit the introduction of the finger, if the uterus could be brought down and fixed, I inserted the mwseux hooks into the neck of the uterus, and brought it down to near the os externum. Giving the hooks to an assistant, I, without any great difficulty, passed my index finger into the cavity of the womb, and found, to my great perplexity, the polypus, which was about the size of a small hen's egg, attached to the fundus and anterior wall of the womb by a pedicle almost the circumference of the polypus itself—say an inch and a half long by three quarters of an inch thick. During the examination the hemorrhage was profuse, until the finger was introduced, which then acted as a tampon, completely arresting the flow, except when from any undue motions of the finger, there would be an irregular dilation of the os with an escape of blood.

What next to do was the perplexing question. It was evident that something had to be done, and that, too, without delay, else our patient would inevitably perish from loss of blood. To attempt to ligate the pedicle, if such it could be called, through an os uteri so slightly dilated, and the cervix filled with the tumor, would have been extremely difficult, if not impossible.

Excision would have been surrounded with the same difficulties, with, perhaps, an increase of the dangers. Evulsion was impossible, with such a pedicle, as I made more than one effort without accomplishing more than having a gush of blood escaping by my finger at every effort.

The idea then suggested itself that I might sever the connection of the pedicle and walls of the uterus with the finger nail: so making tense the portion of the pedicle attached to the anterior wall of the uterus, by depressing the hand and thus forcing the tumor back, I commenced the saw motion with the nail, with firm pressure, and in less time than three minutes, was rejoiced to find that the tissue was giving way under my finger nail. In less time than ten minutes the polypus was completely detached, and without difficulty removed from the cavity of the womb.

After the removal of the tumor, the uterus contracted promptly, and all hemorrhage ceased, so that the tampon, which I fully expected to use, was not necessary. A stimulent and anydyne was now administered, and the patient made comfortable. There was no return of the hemorrhage; nor was the operation followed by any unpleasant symptoms.

The patient rapidly regained her strength, and in a few weeks was able to walk and ride at pleasure.

Six weeks after the removal of the tumor, she menstruated, but not profusely. The two subsequent periods were at the proper time; the discharge, however, was scant, but not attended with any unpleasant symptoms.

The polypus removed was lobulated, and composed of cellular tissue, blood vessels and bundles of fibres, evidently belonging to that variety known as *cellular* polypus.

I do not believe that the pedicle of a true fibrous polypus could be severed in this way. For the removal, then, of this variety, I would suggest an instrument to consist of a thimble which is made to fit, accurately, the index finger, by means of a spring, and so constructed as to carry a concealed blade upon its internal surface, which blade, when the tip of the finger reaches the pedicle to be severed, may be projected by the operator, by means of a wire attached to the blade, and sufficiently long to pass through the os externum.

